# Quality of life under treatment with the immune checkpoint inhibitors ipilimumab and nivolumab in melanoma patients. Real-world data from a prospective observational study at the Skin Cancer Center Kiel

**DOI:** 10.1007/s00432-024-05981-2

**Published:** 2024-10-10

**Authors:** Carolin Grote, Ann-Sophie Bohne, Christine Blome, Katharina C. Kähler

**Affiliations:** 1 University Medical Center Hamburg-Eppendorf (UKE), Hamburg, Germany; 2 University Hospital Schleswig- Holstein (UKSH), Kiel, Germany

**Keywords:** HrQoL, Melanoma, ICI, SF-36, IBDQ-D, Distress thermometer

## Abstract

**Purpose:**

Combined immunotherapy (ipilimumab + nivolumab) has improved survival in stage IV melanoma patients, making Health-related Quality of Life (HrQoL) crucial due to potential immune-related adverse events (irAEs). Previous studies treated HrQoL as secondary/explorative endpoint, and no specific HrQoL questionnaire for melanoma patients on immune checkpoint inhibitor (ICI) therapy exists. This study aimed to gather specific HrQoL data during combined ICI therapy, tracking changes during and after treatment, and examining associations with gender, irAEs, and treatment response.

**Methods:**

35 melanoma patients (22 males, 13 females) undergoing combined ICI were surveyed using the Short-form 36 questionnaire (SF-36), the Inflammatory Bowel Disease Questionnaire – Deutsch (IBDQ-D), and the distress thermometer (DT). HrQoL was evaluated during treatment, after six months, and at the onset of autoimmune colitis.

**Results:**

irAEs occurred in 51.4% of patients, with colitis being the most common (26.1%). 45.7% had progressive disease. SF-36 showed stable HrQoL during treatment and follow-up. Women had worse HrQoL on the physical component scale than men (p = 0.019). Patients with progression showed worse HrQoL over time in physical (p = 0.015) and mental health scales (p = 0.04). IBDQ-D showed constant HrQoL throughout treatment and follow-up. Distress on DT remained constant, with women reporting higher levels of distress.

**Conclusion:**

HrQoL remained stable during and after therapy. Female gender and disease progression negatively impacted HrQoL. The development of irAEs was not associated with HrQoL, though this may not apply to severe irAEs like colitis, which were not assessed.

## Introduction

Malignant melanoma is a tumor that originates from melanocytes in the skin and can result in early lymphatic and blood-borne metastases if left untreated. Melanoma is responsible for more than 90% of skin cancer-related deaths (Garbe et al. 2022). The current therapeutic standard for distant metastatic melanoma is combined immune checkpoint inhibition (ICI) with ipilimumab and nivolumab (Eigentler et al. 2020). There is an increased occurrence of irAEs associated with this treatment, such as autoimmune colitis, which is a crucial and sometimes severe adverse event. Its impact on patients’ HrQoL can be significant (Jackson-Carroll et al. 2024). Symptoms may not only involve pain, weakness, and discomfort but may also result in psychological impairments (Kim et al. 2021).

Whilst ICI has become standard treatment for metastatic melanoma (Carlino et al. 2021), HrQoL data under this treatment remains inadequate (Faury und Foucaud 2020). HrQoL in melanoma patients receiving ICI has been investigated in several large studies, but only as a secondary or exploratory endpoint: CheckMate066, for example, found that patients receiving nivolumab demonstrated improved HrQoL compared to those receiving dacarbazine, with stable *European Organisation for Research and Treatment of Cancer Quality of Life Questionnaire – C30* (EORTC-QLQ-C30) scores over a period of 73 weeks (Long et al. 2016). Furthermore, the Keynote006 trial showed that pembrolizumab resulted in less deterioration of scores compared to ipilimumab, regardless of dose (Daud et al. 2016; Robert et al. 2023). The phase II CheckMate069 trial compared ipilimumab plus nivolumab with ipilimumab monotherapy. HrQoL was maintained in all cohorts (Abernethy et al. 2015). The CheckMate 069 trial compared three cohorts, those receiving the combination of ipilimumab and nivolumab, nivolumab monotherapy and ipilimumab monotherapy. Compared to the ipilimumab cohort, those who received combination therapy and those who received nivolumab monotherapy did not experience reduced HrQoL (Schadendorf et al. 1990).

Although these outcomes are promising, it is important to highlight that there is a lack of systematic research on patients who discontinued treatment due to toxicity. Moreover, the HrQoL data has not been measured against standard samples and is applicable to practical healthcare only to a certain extent due to the strict inclusion and exclusion criteria of clinical trials. As data from clinical trials indicates that ICI therapies maintain stable HrQoL (Chen et al. 2023), research concerning real-world HrQoL data show a different picture. Two studies (Egeler et al. 2024a, b; Pedersen et al. 2023) showed that stage III/IV melanoma patients undergoing adjuvant nivolumab therapy may undergo mental distress during ICI therapy, indicating a potential higher need of support. However, further research in clinical routine is necessary to gain real-world data concerning the long-term effects on HrQoL concerning palliative stage IV melanoma.

Presently, no specific questionnaire is available to measure HrQoL in melanoma patients receiving ICI therapy (Faury und Foucaud 2020). Although established oncology questionnaires such as the EORTC QLQ-C30 are well suited for advanced-stage melanoma patients, the questions are not tailored to ICI therapy, which limits their informative value in the event of adverse events (Machingura et al. 1990; Antoni und Dhabhar 2019). Projects such as the “EORTC advanced melanoma module” (Egeler et al. 2024a, b), the “Monitoring multidimensional aspects of HrQoL after cancer immunotherapy, an open smart digital platform for personalized prevention and patient management” (QUALITOP) (Vinke et al. 2023) or the “functional assessment of cancer therapy—immune checkpoint modulator” (FACT-ICM) (Hansen et al. 2020), which are currently under development, illustrate the great need for clinical data on HrQoL with ICI. These tools were not yet available at the time this study was designed.

The objective of this study was to gather specific HrQoL data of melanoma patients during combined ICI therapy, investigating changes of HrQoL during and after treatment. Secondary endpoints were to identify potential risk factors that could result in a loss of HrQoL. Therefore, the impact of gender, the development of adverse events, and treatment response on HrQoL was examined. They were selected, because previous studies have shown that these factors may have a negative impact on HrQoL (Vogel et al. 2021; Darnell et al. 2020; Kasparian et al. 2009). The study focused additionally on the incidence of autoimmune colitis as a potential side effect, as this study represents a sub-study of a larger project investigating autoimmune colitis in melanoma patients.

## Methods

### Inclusion and exclusion criteria

Inclusion criteria: Patients with metastatic melanoma (cutaneous, uveal, mucosal); palliative therapy with combined ICI (ipilimumab + nivolumab); age over 18 years; signed informed consent form.

Exclusion criteria: Inability to understand the consent form; lack of language skills to understand the consent form and questionnaires; particularly vulnerable persons (e.g. healthy or sick minors, adults unable to give consent, persons in a special dependency relationship).

### Study population

The study protocol was approved by the local ethics committee and conforms to the ethical principles of the Declaration of Helsinki for medical research involving human subjects. Over a period of 14 months, eligible patients undergoing combined immunotherapy with ipilimumab and nivolumab at the Skin Cancer Centre Kiel, Germany, were recruited consecutively during the clinical routine for this prospective observational study.

This study was a sub-study of a larger project whose objective was to characterize gastrointestinal autoimmunity under immune checkpoint inhibition using combined CTLA-4 and PD-1 antibody therapy and correlation with clinical parameters. These parameters included blood analysis, skin microbiome, molecular genetic analysis, stool analysis, gut microbiome, biopsies in the event of autoimmune colitis, food diaries and HrQoL assessment.

Demographic and melanoma-specific data were collected (e.g. gender, age, incidence of irAEs) at each study visit. Clinical data were used to determine the objective response rate (ORR) at weeks 11 and 24 (adding patients in complete and partial remission) and the best overall response (BOR) in accordance with RECIST 1.1 criteria (Schwartz et al. 2016), but without prior definition of a radiographic target lesion. To describe treatment response, the cohort was divided into “progression” (progressive disease) and “no progression” (complete remission, stable disease, partial remission) based on the BOR.

### HrQoL assessment

The HrQoL was evaluated at the initiation of treatment (week (W) 00), during each subsequent infusion appointment (W 03, W 06, W 09), following therapy and associated staging examinations (W 11), after six months regardless of further treatment (W 24), and at the onset of autoimmune colitis (W AE). Due to the lack of an specific questionnaire to measure HrQoL in melanoma patients receiving ICI, HrQoL was assessed using a combination of the generic SF-36 questionnaire (Morfeld et al. 2011), the IBDQ-D questionnaire (Janke et al. 2006) and the distress thermometer (DT) (Mehnert et al. 2006):

The SF-36 it is the most widely used health-related quality of life measure in clinical trials worldwide (Radoschewski 2000) and has been shown to be valid in numerous studies (Tran et al. 2018). In addition, data from several normative samples are available, allowing a reliable comparison of the study population with a healthy population (Morfeld et al. 2011). The SF-36 includes 8 subscales (Physical Functioning, Role Physical, Bodily Pain, General Health, Vitality, Social Functioning, Role Emotional, Mental Health), where higher scores indicate better functioning (range, 0–100). The eight subscales can be assigned to two basic dimensions of subjective health. These two dimensions are presented as component scales for physical (PCS) and mental health (MCS) (t values with mean = 50 and SD = 10, compared to a German norm sample from 1994 (Ellert und Kurth 2004)).

The IBDQ-D was selected as questionnaire with the specific objective of assessing HrQoL in patients with autoimmune colitis, therefore we chose this questionnaire, as this study was a sub-study of a project investigating autoimmune colitis in melanoma patients. It is known from other studies that gastrointestinal side effects, and in particular autoimmune-related colitis, are common with combined immunotherapy with ipilimumab and nivolumab (Som et al. 2019). The IBDQ-D is a validated questionnaire for measuring health-related quality of life in patients with inflammatory bowel disease (Janke et al. 2006) and includes four subscores (bowel, systemic, emotional, and social), measured with a Likert scale, and a total score (sum of subscores) with higher scores indicating better functioning (range, 1–7).

We selected the DT because it is a validated and well-tested measurement tool for patients in oncology (Mehnert et al. 2006), and is part of the clinical standard of care for oncology patients (Donovan et al. 2019). The DT utilizes a visual analog scale (VAS) to indicate the current stress level, ranging from 0 (no distress) to 10 (extreme distress). If the patient’s distress level is five or higher, additional resources such as psychiatry, psychology, or social work should be requested.

### Statistical analysis plan

All data was recorded pseudonymously with Microsoft Excel®, using identification (ID) numbers for patients. The subsequent statistical calculations were carried out using SPSS®. Descriptive statistics were computed for all variables (percentages, frequencies, mean and standard deviation (SD), as applicable). To assess the HrQoL of the entire cohort over time, an analysis of variance (ANOVA) with repeated measures was performed for weeks 0 to 24. A Mauchly test for sphericity was performed. If significance was greater 0.05, sphericity was assumed. If sphericity was violated, significance was determined using Greenhouse–Geisser correction.

The mean values for the factors gender (binary: male vs. female), occurrence of adverse events (irAEs vs. no irAEs), and treatment response (progression vs. no progression) were compared at weeks 0, 11, and 24 using independent samples t tests. Additionally, a repeated measures ANOVA was performed for each of these factors over the course of weeks 0—24. Significance levels equal to or below 0.05 were considered statistically significant.

## Results

### Patient characteristics, demographic and melanoma-specific data

35 participants were enrolled. The study population was 62.9% male and 37.1% female. The average age of the participants was 63 years (SD 13.94). 48.6% (n = 17) of the participants successfully received all 4 infusion doses, while treatment was discontinued in 51.5% (n = 18) of the participants due to several reasons: irAEs occurred in 28.6% (n = 10), 14.3% died (n = 5), and 8.6% (n = 3) experienced disease progression. irAEs were observed in 48.6% (n = 17) of the participants. Among these, the most common was autoimmune colitis (26.1%, n = 6), followed by autoimmune hepatitis, autoimmune pancreatitis, and autoimmune hypophysitis (13% each, n = 3 each). The ORR at week 11 and week 24 remained stable at 35.1% (n = 11). As BOR, 45.7% (n = 16) of patients had progressive disease. 37.1% (n = 13) had partial remission, 8.6% (n = 3) had stable disease and 8.6% (n = 3) had complete remission. Data in week AE (W AE = occurrence of colitis) could only be collected from two of the six patients with colitis. This was due to the colitis of three patients who were being treated in more distant hospitals and to the refusal of one patient to complete the questionnaires. Descriptively, the SF-36 and the IBDQ-D both showed significantly reduced HrQoL values in week AE.

The SF-36 and IBDQ-D were completed by 33 of the 35 patients in week 0. The number of completed questionnaires decreased during the study period, as treatment was discontinued early in 51.5% patients. Fewer questionnaires could be evaluated for the distress thermometer, an important reason for this was that the questionnaire was not digitized after completing and afterwards not archived.

### SF-36

**Fig. 1:**
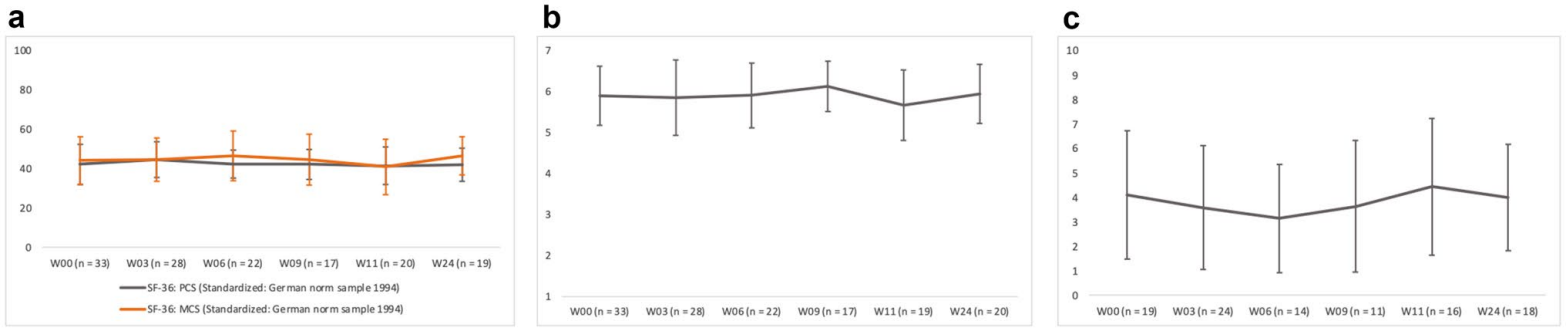
**a** SF-36 componant scale results (with standard deviation) over the course of observation period (range 0–100). *PCS* physical component scale, *MCS* Mental Health Component Scale, *W* Week, *n* number of test subjects evaluated. **b** IBDQ-D total score results (with standard deviation) over the course of observation period (range 1–7). W Week, n number of test subjects evaluated. **c** Distress thermometer VAS results (with standard deviation) over the course of observation period (range 0–10). VAS Visual analog scale, W Week, n number of test subjects evaluated

In the SF-36, HrQoL was consistent on both component scales throughout treatment and follow-up (ANOVA: PCS: p = 0.166, MCS: p = 0.201) (Table 1, Fig. 1a). No deviations were observed below the standard deviation, compared to the German norm sample from 1994, indicating no below-average HrQoL. Also, SF-36 subscales did not show any statistically significant changes over time. The Role Physical and General Health domains showed the lowest values (Role Physical (0–100) W 06: 46.59 (SD 41.04); General Health (0–100) W 06: 47.86 (SD 15.52)), whereas the Social Functioning and Mental Health domains showed the highest values (Social Functioning (0–100) W 06: 79.55 (SD 23.63); Mental Health (0–100) W 06: 73.82 (16,77)). There was an overall (non-significant) decrease in scores at week 11 (at the time of the staging visit) for both the component scales (Fig. 1a) and all subscales (exemplary Role Physical (0–100) W 11: 41.25 (SD 39.96); General Health (0–100) W 11: 41.25 (SD 18.74); Social Functioning (0–100) W 11: 75.0 (SD 25.96); Mental Health (0–100) W 11: 62.8 (SD 20.10)). The female group showed significantly (p = 0.019) lower HrQoL values on the PCS over the time of treatment course than the male group. There was no significant difference between patients with and without irAEs over treatment course, but interestingly, patients without irAEs showed on PCS a significant reduced HrQoL at week 24 (37.44 (SD 7.85); p = 0.028) compared to patients with irAEs (45.82 (SD 8.76)). The cohort with progression showed significant poorer HrQoL values over time for both the PCS (p = 0.015) and the MCS (p = 0.04).

**Table 1:** Questionnaire results using t test and ANOVA

Questionnaires	Independent samples t test						ANOVA	
	week 0		week 11		week 24			
	(n)	p	(n)	p	(n)	p	(n)	p
SF-36								
PCS (overall collective)							8	0.166
MCS (overall collective)							8	0.201
PCS (gender)	13(w)/20(m)	0.908	5(w)/15(m)	0.773	6(w)/13(m)	0.689	3(w)/5(m)	***0.019***
MCS (gender)	13(w)/20(m)	0.432	5(w)/15(m)	0.678	6(w)/13(m)	0.948	3(w)/5(m)	0.973
PCS (irAEs)	16(AE)/17(nAE)	0.847	8(AE)/12(nAE)	0.404	9(AE)/10(nAE)	***0.028***	2(AE)/6(nAE)	0.248
MCS (irAEs)	16(AE)/17(nAE)	0.930	8(AE)/12(nAE)	0.223	9(AE)/10(nAE)	0.470	2(AE)/6(nAE)	0.552
PCS (treatment response)	15(P)/18(nP)	** < *0.001***	6(P)/14(nP)	0.231	5(P)/14(nP)	0.385	2(P)/6(nP)	***0.015***
MCS (treatment response)	15(P)/18(nP)	0.679	6(P)/14(nP)	0.484	5(P)/14(nP)	0.414	2(P)/6(nP)	***0.040***
IBDQ-D								
Total score (overall collective)							8	0.074
Total score (gender)	13(w)/20(m)	0.566	5(w)/14(m)	0.314	6(w)/14(m)	0.404	3(w)/5(m)	0.649
Total score (irAEs)	16(AE)/17(nAE)	0.921	7(AE)/12(nAE)	0.958	10(AE)/10(nAE)	0.696	2(AE)/6(nAE)	0.668
Total score (treatment response)	15(P)/18(nP)	0.185	6(P)/13(nP)	0.063	5(P)/15(nP)	0.713	2(P)/6(nP)	0.163
Distress thermometer								
VAS (overall collective)							4	0.646*
VAS (gender)	7(w)/12(m)	0.354	4(w)/12(m)	0.883	5(w)/13(m)	***0.049***	2(w)/2(m)	0.769*
VAS (irAEs)	8(AE)/11(nAE)	0.844	10(AE)/6(nAE)	0.523	9(AE)/9(nAE)	0.677	2(AE)/2(nAE)	0.06*
VAS (treatment response)	8(P)/11(nP)	0.754	5(P)/11(nP)	0.373	4(P)/14(nP)	0.616	1(P)/3(nP)	0.863*

Results of the t-test and ANOVA with repeated measures for the SF-36, the IBDQ-D and the distress thermometer: Performance of t-test for week 0, week 11 and week 24 for the subgroups gender, occurrence of irAEs and treatment response. The ANOVA with repeated measures was performed for the entire collective and the three subgroups. Significances marked bold and cursively

*n* number of test subjects evaluated, *p* p-value, *w* female, *m* male, *AE* irAEs, *nAE* no irAEs, *P* progression, *nP* no progression, *ANOVA* analysis of variance *MCS* mental health component scale, *PCS* physical component scale *ANOVA not applicable due to too few test subjects

### IBDQ-D

The total score for IBDQ-D remained constant throughout the treatment weeks (exemplary W 06: 5.90 (SD 0.79); W 24: 5.93 (SD 0.72)) and did not show significant differences in mean values over time (p = 0.074) (Fig. 1b). Week 11 also recorded the lowest value for this questionnaire (total score W 11: 5.66 (SD 0.86)). Over time, the “Bowel” and “Social” subscores showed the highest values (Bowel W 06: 6.21 (SD 0.75); Social W 06: 6.29 (SD 0.94)), while “Systemic” showed the lowest (Systemic W 06: 5.19 (SD 0.98)). Gender, irAEs, and treatment response evaluations did not yield significant differences in the IBDQ-D (Table 1).

### Distress thermometer

Results from the DT consistently indicated acute distress with values less than 5 on the VAS in all patients, indicating no need for psycho-oncological intervention at any point. The level of distress remained constant throughout the therapy period (exemplary VAS W 06: 3.14 (SD 2.21); VAS W 24: 4.0 (SD 2.16)) (Fig. 1c); an ANOVA could not be run due to few test subjects. Descriptively the female group reported a higher level of distress over the course of treatment. However, the t test for independent samples reached statistical significance only at week 24 (p = 0.049; female VAS W 24: 5.6 (SD 2.07); men VAS W 24: 3.38 (SD 1.94)). No significant differences in DT were observed based on the occurrence of irAEs and treatment response (Table 1).

### Endpoints

The HrQoL of the cohort remained constant throughout the observation period. There was no deterioration during treatment or in the following months. Nevertheless, the overall HrQoL was already reduced at the start of treatment, which can be attributed to the patients’ advanced melanoma disease. The factors female sex and development of progression showed a negative influence on HrQoL in SF-36 and DT. The development of irAEs did not influence HrQoL in this study.

## Discussion

As ICIs have been shown to significantly improve the survival of melanoma patients, HrQoL is an important factor. Previous trials have shown that ICI therapy has little or no impact on HrQoL, but the occurrence of irAEs could lead to a significant loss of quality of life (Schulz et al. 2022). Questionnaires play therefore an important role in assessing HrQoL.

In order to obtain the most accurate HrQoL possible, several measurement instruments were combined, following the procedure used in previous studies (Holterhues et al. 2011): The SF-36 questionnaire was employed in this study, providing valid results that were easy to interpret due to its widespread use in previous studies (Tran et al. 2018; Boutros et al. 1990; O’Reilly et al. 2020; Reinhardt et al. 2023). The comparison with a norm sample was beneficial since there is limited HrQoL data available for melanoma patients in comparison to a healthy population (O’Reilly et al. 2020). The IBDQ-D was not identified in the literature search for melanoma patients or ICI-mediated colitis, and unfortunately proved to be impractical to use, as there were often misunderstandings among patients during its administration. However, as the results were largely consistent with those of the SF-36, we assumed that they reflected the HrQoL of the cohort. The distress thermometer also provided comparable results. Thus, the combination of several questionnaires proved to be feasible, to obtain confirmatory results. Although the initial questions could be answered, the use of three different questionnaires is complicated and time-consuming for both patients and investigators. It is evident that with the increasing number of melanoma patients undergoing ICI therapy each year (Carlino et al. 2021), this patient population requires a specific questionnaire (Vogel et al. 2021).

### The questionnaires

The SF-36 did not show a significant change in HrQoL over the weeks. Strikingly the lowest scores were measured at week 11 for both component scales and subscales although these results did not reach statistical significance. This could be explained by small sample size and further observations are needed to verify this observation. One possible explanation could be that patients were experiencing distress and discomfort prior to discussing the results of the staging examinations at the end of treatment. Another possibility is that the four cycles of therapy were a cumulative burden for the patients. In this trial, the study population reported poorer HrQoL than the normative sample at all time points in the SF-36. However, there was an improvement from week 11 to week 24. Other studies suggest that melanoma survivors (Vogel et al. 2017) and long-term survivors after treatment with ipilimumab do not have a significantly worse HrQoL in the long-term than a healthy population (Egeler et al. 2023). Follow-up in those studies was up to three years. Therefore, it can be assumed that the subjects observed here will continue to experience an improvement in HrQoL over time. The two patients who completed the SF-36 in week AE, i.e. at the onset of colitis, had scores below the standard deviation. Mental well-being was less affected than physical well-being. Although this observation in two patients does not allow a general statement, the results suggest that the development of autoimmune colitis, which led to hospitalization in both cases, particularly affected the somatic level of HrQoL.

The lack of comparative studies made interpretation of the IBDQ-D difficult. Nevertheless, the results were largely consistent with those of the SF-36: Mean scores on the IBDQ-D remained constant over time, but HrQoL scores were generally good (≥ 5). Although it is recommended that only the total score be used in clinical trials, the descriptive results of the subscores were considered. The good results in the “Bowel” score can be explained by the absence of gastrointestinal complaints in most patients. The positive assessment in the “Social” score is in line with the SF-36 observations, where there was also a high assessment of social functioning. At week AE, there was a decrease in all subscores except “Emotional” which remained almost constant. The “Systemic” score fell sharply, but “Bowel” and “Social” also showed significantly lower scores. This is not surprising given the hospitalization of the patients with colitis. Interestingly, the symptoms had less of an impact on emotional well-being, which is consistent with the SF-36 results, where mental well-being was least impaired at week AE. Although the subjects were provided with precise information beforehand, the IBDQ-D questionnaire caused irritation among the patients. Some comments indicated that the questionnaire was not relevant to the person completing it. Some participants declined to complete the questionnaire, arguing that they did not have chronic inflammatory bowel disease.

The distress thermometer showed an average distress level lower than five and remained constant throughout the treatment and follow-up period. There was no acute need for intervention at any time. Few patients reported receiving psychological or psychiatric care, and the need for psychopharmacological support was low. These observations are in line with other studies: In one trial, melanoma did not cause severe psychosocial distress (Loquai et al. 2013). Another study found a low need for psycho-oncological support among melanoma patients on immune checkpoint inhibition, both at baseline and during treatment (Wiens et al. 2021). A larger study suggests that patients undergoing systemic therapy have a lower need for psycho-oncological support, while an increased need may arise after completion of therapy (Forschner et al. 2017). This observation reflects the experience in clinical practice that patients feel well supported during ongoing systemic therapy, while the need for support may increase after completion of therapy and fewer clinic visits. However, a recent review showed that women, younger patients, and patients with a lower level of education who undergo chemotherapy or immunotherapy are significantly more likely to experience anxiety and depression, particularly during the treatment period (Kungwengwe et al. 2024). The authors of this review recommend enhanced psychooncological support during therapy and to implement long-term studies concerning depression and anxiety in future research.

### Identifying risk factors

The study population consisted of 35 participants, including 22 males and 13 females. Other studies have shown that there are differences in HrQoL according to biological sex (Egeler et al. 2023). Therefore, a targeted evaluation of this aspect was conducted. Evaluation of the SF-36 by gender showed a significant difference in the PCS, with the male cohort performing slightly better. The DT showed higher distress levels in women, but only significantly at week 24. Autoimmune adverse events occurred in approximately half of the subjects, with colitis being the most common. The analysis of HrQoL showed no significant differences between patients with and without irAEs. Seven patients died during the observation period, mainly due to melanoma progression. Treatment response rates were significantly lower compared to other studies (Hodi et al. 2018), possibly due to the small number of cases and other assessment methods. Disease progression had a negative impact on HrQoL in SF-36 and DT, especially in patients with later progression. The IBDQ-D scores did not show any significant differences regarding gender, occurrence of irAEs or treatment response.

### Future prospects

From these results, it can be concluded that more psycho-oncological support and attention should be given to women and people who are currently experiencing a worsening of their disease in routine clinical practice. The development of irAEs must be viewed in a differentiated way. Not every side effect affects the HrQoL. However, serious side effects are likely to play an important role. This should be confirmed by further real-world studies with a larger number of cases. As the prognosis of melanoma patients continues to improve, in part due to treatment with ICI (Bolick und Geller 2021), monitoring the HrQoL of patients with advanced melanoma is becoming increasingly important (Merrick et al. 2024). Developments such as the QUALITOP (Vinke et al. 2023) or the FACT-ICM questionnaires (Hansen et al. 2020) are providing important impetus in this area of research and give hope that the HrQoL of this special group of patients will be better measured in the future.

All questionnaires used in this study do not yet exist in digital form. But the assessment of HrQoL in daily clinical practice requires practical solutions, especially due to time constraints and possible errors by untrained staff. One promising solution is the digitization of questionnaires that can be completed on tablets. Not only is it easy for patients to use, but it also allows for immediate storage and easier analysis of data in large databases. Studies in the field of telemedicine are already exploring the application-driven collection of HrQoL data (Knapp et al. 2021). The proliferation of digital platforms where patients share their experiences underlines the urgency of digitization in this area. In the future, artificial intelligence combined with real-world data could also contribute to individualized therapy determination and support to improve patients’ HrQoL and simplify disease management (Cotté et al. 2020; Guerrisi et al. 2022).

### Limitations

This study had limitations: It is an exploratory study at a single skin cancer center; a study with a significantly larger number of cases would be needed to confirm the results. Furthermore, the lower number of patients can be explained by the fact that this is a sub-study of a project on autoimmune colitis, which resulted in limitations due to the additional requirements of the main project. There was a significant reduction in the number of patients due to missing data when evaluating the characteristics of gender, adverse events, and treatment response. Therefore, the data from these cohorts should be considered as indicative. Upon reflection, it appears that the IBDQ-D questionnaire was not the most suitable choice for this patient population. Despite providing precise instructions to subjects beforehand that the questionnaire was intended for patients with IBD, it caused irritation among patients. A questionnaire such as the PRO-CTCAE might have been more appropriate (Da Silva Lopes et al. 1990). Another limitation is the week AE. A subject number of 2 allows only a descriptive observation of the results. As in other studies, it was not possible to specifically follow up patients when severe irAEs leading to hospitalization occurred. The use of application-guided questionnaires could be of great benefit in the future for this purpose.

## Conclusion

In this study, the HrQoL of melanoma patients receiving combination immunotherapy was assessed using the SF-36 questionnaire, the IBDQ-D and the distress thermometer. The aim was to monitor HrQoL during the course of therapy and up to six months after the start of therapy by collecting real-world data and to determine whether gender, treatment response and development of irAEs had an influence on HrQoL. In addition, HrQoL was to be specifically monitored in the event of the development of colitis. It was shown that the cohort’s HrQoL was unaffected by therapy and did not change during the follow-up period. However, the cohort showed a poorer HrQoL than a healthy population. Female gender and disease progression may be considered risk factors for a reduced HrQoL. The development of irAEs did not influence HrQoL in this study, but it is reasonable to assume that severe irAEs, such as the development of colitis, lead to a deterioration in HrQoL. These results may be relevant for routine clinical practice in the future. Therefore, it would be useful to have a questionnaire that is disease- and therapy-specific, and thus better captures HrQoL when irAEs occur.

## Data Availability

No datasets were generated or analysed during the current study.

## List of abbreviations

AE: Adverse event
ANOVA: Analysis of variance
BOR: Best overall response
CTLA-4: Cytotoxic T-lymphocyte-associated protein 4
DT: Distress thermometer
EORTC-QLQ-C30: European Organisation for Research and Treatment of Cancer Quality of Life Questionnaire–C30
HrQoL: Health-related quality of life
ICI: Immune checkpoint inhibitor
irAEs: Immune related adverse events
MCS: Mental health component scale
ORR: Objective response rate
PCS: Physical component scale
PD-1: Programmed death-1
SD: Standard deviation
SF-36: Short-form 36 questionnaire
VAS: Visual analog scale
W: Week
